# Variation in energy sorghum hybrid TX08001 biomass composition and lignin chemistry during development under irrigated and non-irrigated field conditions

**DOI:** 10.1371/journal.pone.0195863

**Published:** 2018-04-23

**Authors:** Brian A. McKinley, Sara N. Olson, Kimberley B. Ritter, Dustin W. Herb, Steven D. Karlen, Fachuang Lu, John Ralph, William L. Rooney, John E. Mullet

**Affiliations:** 1 Department of Biochemistry and Biophysics, Texas A&M University, College Station, Texas, United States of America; 2 Department of Energy Great Lakes Bioenergy Research Center, United States of America; 3 Department of Crop and Soil Science, Oregon State University, Corvallis, Oregon, United States of America; 4 Department of Biochemistry, the Wisconsin Energy Institute, University of Wisconsin, Madison, Wisconsin, United States of America; 5 Department of Soil and Crop Sciences, Texas A&M University, College Station, Texas, United States of America; Louisiana State University College of Agriculture, UNITED STATES

## Abstract

This study was conducted to document the extent and basis of compositional variation of shoot biomass of the energy *Sorghum bicolor* hybrid TX08001 during development under field conditions. TX08001 is capable of accumulating ~40 Mg/ha of dry biomass under good growing conditions and this genotype allocates ~80% of its shoot biomass to stems. After 150 days of growth TX08001 stems had a fresh/dry weight ratio of ~3:1 and soluble biomass accounted for ~30% of stem biomass. A panel of diverse energy sorghum genotypes varied ~6-fold in the ratio of stem structural to soluble biomass after 150 days of growth. Near-infrared spectroscopic analysis (NIRS) showed that TX08001 leaves accumulated higher levels of protein, water extractives and ash compared to stems, which have higher sugar, cellulose, and lignin contents. TX08001 stem sucrose content varied during development, whereas the composition of TX08001 stem cell walls, which consisted of ~45–49% cellulose, ~27–30% xylan, and ~15–18% lignin, remained constant after 90 days post emergence until the end of the growing season (180 days). TX08001 and Della stem syringyl (S)/guaiacyl (G) (0.53–0.58) and ferulic acid (FA)/*para*-coumaric acid (*p*CA) ratios were similar whereas ratios of *p*CA/(S+G) differed between these genotypes. Additionally, an analysis of irrigated versus non-irrigated TX08001 revealed that non-irrigated hybrids exhibited a 50% reduction in total cell wall biomass, an ~2-fold increase in stem sugars, and an ~25% increase in water extractives relative to irrigated hybrids. This study provides a baseline of information to help guide further optimization of energy sorghum composition for various end-uses.

## Introduction

World population growth and development projected by 2050 will significantly increase the demand for food, feed, energy, chemicals, and bio-based products [1]. Renewable low-cost sources of plant-derived biomaterials could significantly enhance long-term food, energy, and environmental security. High-biomass C4 grasses such as *Sorghum bicolor*, *Miscanthus x giganteus*, sugarcanes (*Saccharum* spp.), and *Pennisetum* genotypes (i.e., Napier grass) can accumulate >40 Mg of dry biomass per hectare each growing season [2,3]. Sugarcane, the most economically important high-biomass C4 grass, was grown on 26 million ha and produced 1.83 billion Mg of high-moisture stem biomass in 2012 [4]. Sugarcane grown in Brazil provides an economical source of sucrose, bio-power, and bioethanol supplying a large portion of Brazil’s transportation fuel [5]. In the U.S., bioethanol production from corn (*Zea mays*) grain has increased 10-fold since 2000 to nearly 15 B gallons/year consuming 30–40% of the corn grain crop [6]. Corn bioethanol production has an energy output/input ratio of 1.4–2.3 whereas this ratio is significantly higher for energy crops such as sweet sorghum (~21), sugarcane (~8–10) and Miscanthus (~22) [7]. The US Energy Independence and Security Act of 2007 mandated that ~30% of the fuel used for US transportation be met by production of alternative fuels by 2030, with no more than 15 billion gallons being derived from grain crops and ~21 billion gallons from lignocellulose and other non-grain sources of biofuels [8,9]. Progress towards the latter goal has been slow due to the high cost of biofuels production from lignocellulosic biomass. Highly productive energy crops that accumulate low-cost biomass with a composition optimized for harvesting, storage, processing, and conversion are needed to produce cost-competitive biofuels and bio-products [10].

C4 grasses are excellent genetic systems for the design of next-generation high-biomass multiuse energy crops due to their photosynthetic efficiency, high biomass yield potential, and wide adaptation [3,7,11,12]. Energy sorghum is unique among the high-biomass C4 grasses because sorghum is an annual hybrid crop [3,13]. Energy sorghum’s drought resilience, good water use efficiency, heat tolerance, and low input requirements allows production on annual cropland that is marginal for most food crops [3]. The sorghum germplasm collection (n = 43,000) contains extensive genetic and phenotypic diversity for traits relevant to the design of high-biomass energy crops [13]. Sorghum’s facile genetics, well established energy sorghum hybrid breeding and production systems [13], good genomic resources, and rapidly improving technology for *cis*-genics and genome editing [14–16], make it an excellent genetic platform for fundamental research on C4 grass grain, forage, and bioenergy crops and a source of commercial hybrids for bio-based industries.

Cell walls that comprise the bulk of plant biomass evolved to provide mechanical strength and protection from pests making them recalcitrant to the release of sugars for microbial upgrading [17]. In grasses, the structural portion of biomass is composed primarily of cellulose, glucuronoarabinoxylan (GAX) hemicelluloses, pectins, and lignin, together with low amounts of wall-associated proteins, inorganics, and other compounds. Cellulose is the most abundant cell wall polysaccharide and represents the largest potential source of glucose for microbial production of biofuels and bio-products. GAX, a complex hemicellulosic polysaccharide, has a xylan backbone decorated with (4-*O*-methyl)glucuronic acid, and arabinose substituents, and hydroxyl groups that may be acetylated [18]. GAX is also covalently linked to the lignin polymer matrix through ferulate substituents that are attached to the primary C5-OH of arabinosyl units [19]. GAX deconstruction is complex requiring expensive enzymatic cocktails, and the sugars released are not readily metabolized by most yeast strains [20]. The constituents of the cell wall include an amorphous lignin polymer matrix that increases cell wall strength and recalcitrance. The soluble portion of plant biomass is comprised of sugars, proteins, amino acids, inorganics, water-extractable organic acids, phenolic glycosides, alditols, and mixed-linkage glucans [21]. The water-insoluble but ethanol-extractable fraction of biomass is composed of lipids, waxes, terpenoids, and other hydrophobic compounds such as chlorophyll.

Comprehensive knowledge of the composition of biomass accumulated by bioenergy crops under field conditions is needed for the design of optimal systems for production, harvesting, storage, and biorefinery operations [22–26]. NIRS is routinely used for high-throughput analysis of biomass composition. The National Renewable Energy Laboratory (NREL) created an NIRS prediction model to facilitate the analysis of biomass composition [27]. This model is capable of predicting the relative abundance of cellulose, lignin, xylose, arabinose, and galactose as well as hot-water-soluble extractives, hot-ethanol extractives, ash, and protein [27,28]. NIRS analysis of total shoot biomass from diverse sorghum genotypes used for forage, grain, sugar and biomass production revealed that sorghum germplasm has a wide compositional range [28]. Part of this variation is due to sorghum genotypes that accumulate high levels of sucrose in stems during the reproductive phase [29–32].

First-generation energy sorghum hybrids such as TX08001 were developed using a unique breeding program that enables production of late flowering energy sorghum from early flowering inbreds [13]. TX08001 has been characterized under field conditions for biomass yield, phenology, radiation use efficiency, nitrogen use efficiency and in multi-location field studies [23,33,34]. Additional information about variation in energy sorghum hybrid composition under field conditions is needed to optimize harvesting, processing, and conversion of derived biomass to biofuels and other bioproducts and to guide further improvements in energy sorghum biomass composition. In the current study NIRS and NMR methods were used to characterize the composition of TX08001 grown in irrigated and non-irrigated field conditions.

## Materials and methods

### 1. Harvest of the Energy Sorghum Association Panel (ESAP)

Harvest of plant tissue for the analysis of energy sorghum composition from the energy sorghum [*Sorghum bicolor* (L.) Moench] association panel (ESAP) was conducted at the Texas A&M University Field Station near College Station, Texas (30°37'40˝N, 96°20'3˝W, 100 m above sea level) during the summer of 2012 using previously described fertilization, planting densities, and plot layout [33]. At this location, soils are a Belk Clay (fine, mixed, thermic Entic Hapludert) [35] that can hold up to 40% water by volume [36]. Rows were thinned to 10 cm spacing and the spacing between rows was 76 cm, resulting in a planting density of 132,000 plants per hectare. Five plants were harvested from the center of the row to avoid edge effects. Five adjacent plants were harvested to mitigate unintentional selection. Compositional analysis was confined to a three internode section with the middle internode of the three internode section located at the mid-point of the stem. Harvesting of internode sections located at the middle of the stem was performed to minimize variation in composition due differences in stage of internode development. Internode samples from 3 plants were excised from each plant and bulked to form one sample per genotype. The bulked stem sections were cut into smaller pieces and subsequently dried in a forced air oven at 60°C. Internode sections were ground in a Wiley Mill (Thomas Scientific, Inc.) until the biomass particles could pass through a 2 mm sieve and used for NIRS analysis. To prepare internode tissue for analysis of MLG and nonstructural carbohydrates, biomass was ground further in a Cyclone Sample Mill (Udy Corporation, Fort Collins, Colorado, USA) until the tissue exhibited the consistency of a powder.

### 2. Harvest of the energy sorghum hybrid: TX08001

The composition of the energy sorghum hybrid TX08001 was characterized during development in the field in 2008 and 2009 at the Texas A&M University Field Station near College Station, Texas. Plants were grown in the same location and at similar densities as the ESAP described above. The experimental design for collection of TX08001 biomass from sorghum plants grown under field conditions was previously described [32,33].

### 3. Harvest of stem tissue from the sweet sorghum Della

Harvest of sweet sorghum tissue for stem composition analysis was conducted in 2012. Plants were grown at the same location as the ESAP and TX08001 experiments. Fertilizer was applied at the same rate as the other two experiments. Plots were thinned to 10 cm spacing. At the time of harvest, 9 plants were harvested. Plants were harvested near the middle of the row away from gaps in plant density. Three adjacent plants were selected from each row to maintain random sampling. Leaf tissue was removed from the stem. Internode tissue from three fully elongated mid-stem internodes was excised at the node. These internodes were cut into smaller pieces to facilitate drying and subsequently dried in a forced air oven at 60°C. Internodes sections were ground using identical methods as stated for samples from TX08001 and the ESAP.

### Near-infrared spectroscopic analysis of internode composition

To prepare ground tissue for NIRS analysis, the ground internode samples were re-dried at 60°C to remove residual moisture thereby minimizing variation in moisture content between samples. NIR spectra were acquired using the stationary module of a Foss XDS grating-monochromator (Foss North America, Eden Prairie, MN). The samples were scanned in reflectance mode across the wavelength range of 4,000–9,000 cm^-1^. Two NIR spectra of each sample were collected on separate occasions to ensure reproducibility of measurement. The spectra were analyzed using a previously published compositional prediction model developed for sorghum by NREL [27]. The NREL NIRS model was originally constructed using whole plant biomass. To assess the applicability of the NIRS model for predicting the composition of stems and leaves independently, Global H (GH) values were assessed. Spectra with GH >3 are generally considered too dissimilar to the global spectra set to have their composition accurately predicted by the model. Analysis of the GH values of all TX08001 internode samples revealed an average GH = 1.26, with minimum and maximum GH values of 0.521 and 2.72 respectively, indicating that the NREL NIRS model can be used to predict the composition of leaves and stems. Additionally, the variation between TX08001 leaf and stem composition and the composition of the whole plant is much lower than the variation in composition observed between grain, energy and sweet sorghums that were used to train the NREL NIRS model.

### Soluble carbohydrate quantitation

To quantify the abundance of stem non-structural carbohydrates, internode and leaf tissue was ground to a small particle size in a Cyclone Sample Mill (Udy Corporation, Fort Collins, Colorado, USA) followed by removal of residual moisture overnight using a forced air oven at 70°C. Next, 200 mg of finely ground biomass was weighed (± 0.5 mg) using an analytical balance and transferred to a 15 mL conical glass tube. Water-soluble NSCs were extracted in 10 mL of water/sodium azide (200 mg/L) solution at 50°C for 48 h with agitation. This length of incubation was experimentally determined to be optimal for this experimental set-up. This extraction time allowed the extraction solvent to fully penetrate all of the biomass particles and extract the soluble carbohydrates. Aliquots of 20 μL were diluted 50X into 980 μL of deionized water. All HPLC samples were filtered using 0.45 μm cellulose acetate sterile filters. Concentrations of sucrose, glucose, and fructose and glucose released from starch digestion were quantified using high-performance anion-exchange-pulsed amperometric detection (HPAE-PAD) with a Carbopac PA1 analytical column (Dionex, Sunnyvale, CA, USA) as well as a Borate trap (Dionex) and an Aminopac column (Dionex) to reduce borate and amino acid interference. A solution of 75 mM NaOH was made from 50% NaOH solution (Sigma-Aldrich) to eliminate carbonate contamination. The NaOH eluent was vacuum-degassed overnight and stored under a helium atmosphere for the duration of the chromatographic run. The standard curve was validated by the incorporation of curve-validation samples of known concentration throughout the experiment in accordance with the NREL Laboratory analytical procedure [37]. Mixed-linkage glucan (MLG) was quantified using the Megazyme β-glucan (mixed linkage) assay kit, assay procedure A (Megazyme, Bray, Ireland). Glucose released from MLG digestion was assayed using the glucose oxidase/peroxidase reaction and absorbance was determined using a Beckman Coulter DU730 Life Sciences UV/Vis spectrophotometer (Beckman Coulter, Brea, CA).

### Nuclear magnetic resonance characterization of cell-wall composition

The whole-cell-wall gel HSQC spectra were acquired following the standard protocol [38,39]. The following protocol was performed on two single plant biological replicates of TX08001 at 150 DAE from experiment 2 and Della harvested at anthesis (80 DAE) from experiment 3. Briefly, extract-free course-ground biomass (300 mg) was milled to a fine powder with a Fritsch Pulverisette 7, using ZrO_2_ grinding jars (20 mL) and 10 x 10 mm ZrO_2_ balls at 600 rpm, 15 cycles (5 min on, 10 min rest). The fine powder (40 mg) was transferred to a 5 mm NMR tube and gelled with a mixture of DMSO-d_6_/pyridine-d_5_ (4:1, 500 μL). The whole-cell-wall gel was characterized by HSQC spectroscopy on a Bruker Biospin (Rheinstetten, Germany) Avance 700 MHz NMR spectrometer equipped with a gradient 5 mm QCI ^1^H/^31^P/^13^C/^15^N cryoprobe with inverse geometry (proton coils closest to the sample). The central DMSO solvent peak was used as internal reference (δ_c_ 39.5, δ_H_ 2.49 ppm).

### Analysis of lignin composition

The chemical composition of the lignin was determined by Derivatization Followed by Reductive Cleavage (DFRC) [40,41]. This analysis was conducted on two single plant biological replicates of both TX08001 at 150 DAE from experiment 2 and Della harvested at anthesis (80 DAE) from experiment 3 The DFRC analysis was performed as described [42]. In a 2 dram vial with a stir bar and pressure-release PTFE cap (60 psi limit), dry extract-free whole cell walls (45–50 mg) were treated with a solution of acetyl bromide in acetic acid (1:4, v/v, 5 ml) at 50°C for 3 h. The solvents were removed on a SpeedVac concentrator (ThermoFisher Scientific, 50°C, 30 min, 1.0 torr). The wet film was treated with ethanol (200 proof, 1 mL) to quench any remaining acetyl bromide, and then the ethanol was removed on a SpeedVac (50°C, 10 min, 1.0 torr). The samples were then immediately suspended in a solution of 1,4-dioxane: acetic acid: water (5:4:1, by volume, 5 mL), and zinc nano-powder (150 mg) was added to the vial. The reaction was stirred for 3 h at room temperature with the addition of more zinc as required to keep a fine suspension. The reaction was then quenched with saturated ammonium chloride, spiked with a recovery standard diethyl 5,5'-diferulate (34 μg). The organics were extracted with dichloromethane (5 × 4 mL), the combined organic fractions were dried over sodium sulfate, filtered, and the solvent was removed under vacuum. The reactive free hydroxyl groups were acetylated with a mixture of acetic anhydride and pyridine (1:1, 4 mL, 12 h). The excess acetic anhydride and pyridine was removed on a rotary evaporator. The crude film was dissolved in ethyl acetate (200 μL) and then diluted with hexanes (200 μL). This caused the residual sugars to precipitate from the solution. The ethyl acetate: hexanes solution was loaded on to a Supelco Supelclean solid-phase extraction (SPE) tube (150 mg LC-SI, Sigma-Aldrich part #505048). The products were eluted with ethyl acetate: hexanes (1:1, 8 mL), the organics were combined and the solvent was removed on a rotary evaporator. The resulting dry film was transferred to a GC vial with dichloromethane (1 mL), the vial was spiked with an injection standard 1,1-bis-(4-phenol)ethane (BPA, 50 μg) and injected into a Shimadzu TQ8030 GC-MRM-MS for quantitative analysis. Instrument calibration was performed using synthetic standards.

### Statistical analyses

Mean comparisons were conducted using the two tailed t-test. This was implemented using the TTEST() function in Excel.

## Results

### Variation in stem biomass composition among TX08001 and high biomass inbreds

**Fig 1:**
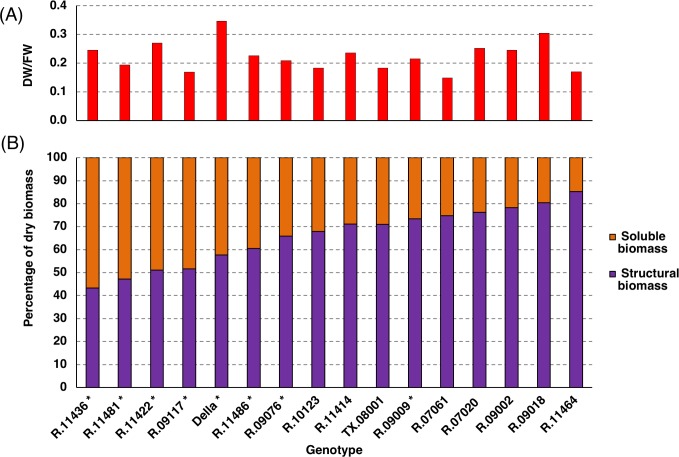
Variation in sorghum stem dry biomass composition in a representative survey of energy and sweet sorghums. (A) Ratio of dry biomass to fresh biomass of a diverse selection of sorghum internodes at 150 DAE (experiments 1, 2, and 3, see Materials and Methods). (B) NIRS prediction of the percentage of the sorghum stem dry biomass that is composed of soluble and structural molecules of the ESAP in 2012 at 150 DAE (experiment 1). Accessions with (*) were at or past anthesis developmentally. Each bar represents data obtained from five bulked internode segments from ESAP accessions. Soluble and structural compositional data was obtained from the NIRS prediction model. Della and TX08001 stem segments from 150 DAE (experiments 2 and 3), which are the average of nine plants, are included in the figure for comparison.

**Fig 2:**
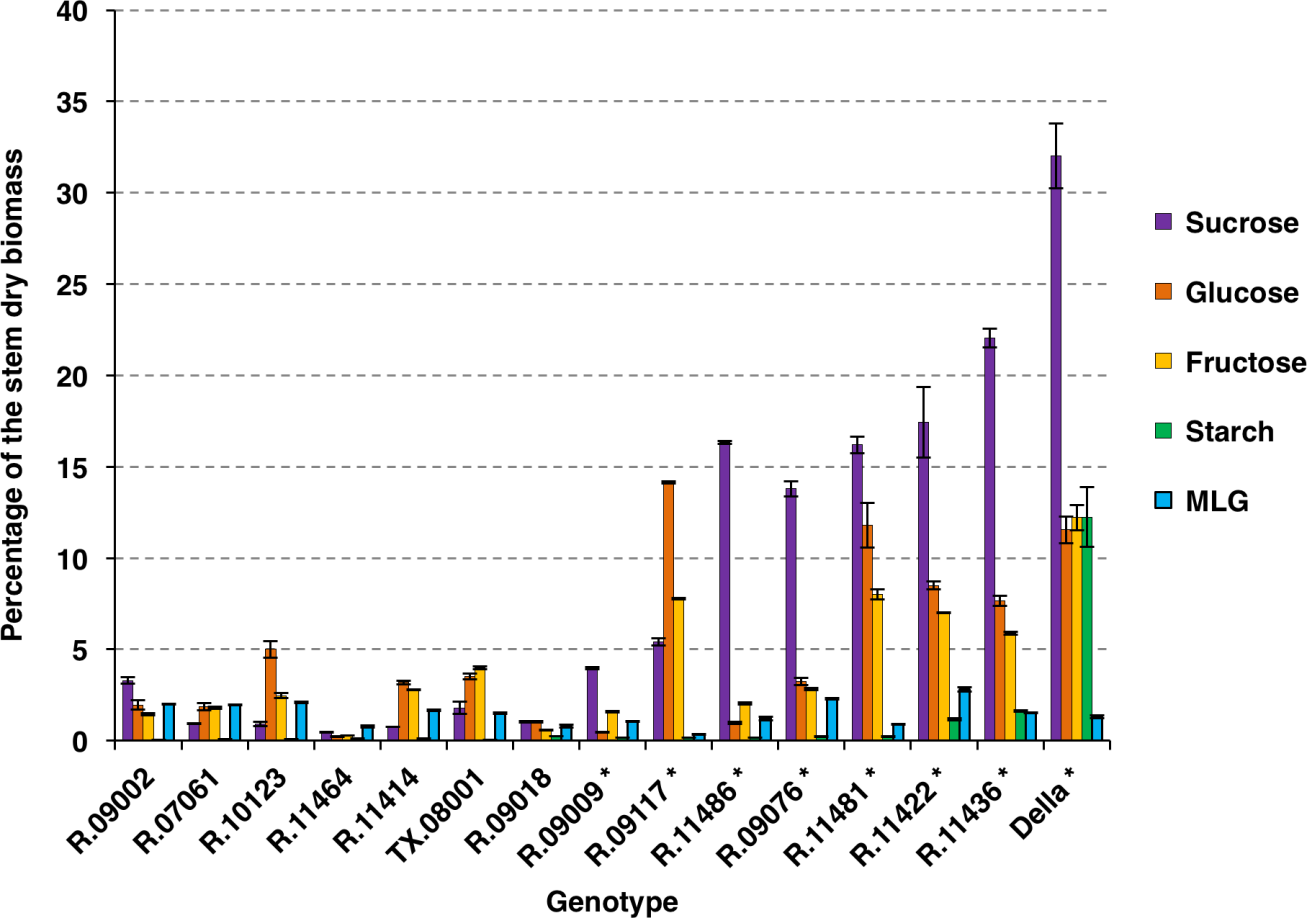
Nonstructural carbohydrate profiles of sorghum stems from a selection of representative energy and sweet sorghums as a percentage of the dry biomass of the stem. Data were obtained from plant material from experiments 1, 2, and 3. ESAP samples were obtained from bulked internode samples of five plants that were harvested at 150 DAE (experiment 1). Data from TX08001 (experiment 2) and Della (experiment 3) were obtained from plants harvested at 150 DAE and 80 DAE respectively and are from 9 biological replicates bulked into three samples. Accessions with (*) were at or past anthesis developmentally. Measurement of sucrose, glucose, fructose and starch was performed in duplicate and MLG assays were performed in triplicate. Error bars represent standard error of the mean.

Energy sorghum hybrids were grown in College Station for ~150 days. At the time of harvest many of the energy sorghum accessions remained in the vegetative phase whereas some had transitioned into the floral development stage. The accumulated stem biomass had a dry/fresh weight ratio of ~0.17 and soluble compounds accounted for ~30% of the biomass (Fig 1A and 1B and S1 Fig). TX08001 was compared to sorghum inbreds that were previously found to be useful for high biomass production to assess the extent of variation in these stem traits among a selection of energy sorghum germplasm [13]. The dry/fresh weight ratios of stems of these materials ranged from ~0.15 to 0.35 at harvest. Analysis of dry biomass composition showed that, among these genotypes, the soluble fraction of stem biomass ranged from ~15% to ~55% of total dry biomass. The relative amount of soluble biomass was not correlated with percent dry weight (PCC = 0.13). However, genotypes that had reached anthesis by 150 days after emergence (DAE) tended to have higher relative amounts of soluble biomass. Sorghum genotypes are known to accumulate stem sucrose following floral initiation [29,31]. Therefore, a selection of a subset of the energy sorghum accessions from the diversity panel had reached or passed anthesis by 150 DAE (Fig 2 and S1 Fig, denoted by *) and other accessions that were still vegetative (Fig 2 and S1 Fig, no asterisks) were analyzed for stem sugar accumulation. As expected, genotypes that had reached anthesis prior to harvest had higher levels of stem sucrose compared with TX08001 and other genotypes that had not flowered. Mixed linkage glucans (MLG) were also assayed since these polymers can be readily extracted and digested contributing to fermentable carbohydrate yield. All genotypes exhibited measureable levels of mixed linkage glucan in their stems, varying between 1–3%. The abundance of MLG was not correlated with developmental stage or with increasing levels of other stem carbohydrate pools (Fig 2 and S1 Fig). Significant variation in the relative abundance of sucrose, glucose, fructose, and starch was observed in stems of genotypes that accumulated high levels of these compounds (Fig 2 and S1 Fig).

### Biomass accumulation in TX08001 energy sorghum hybrids

**Fig 3:**
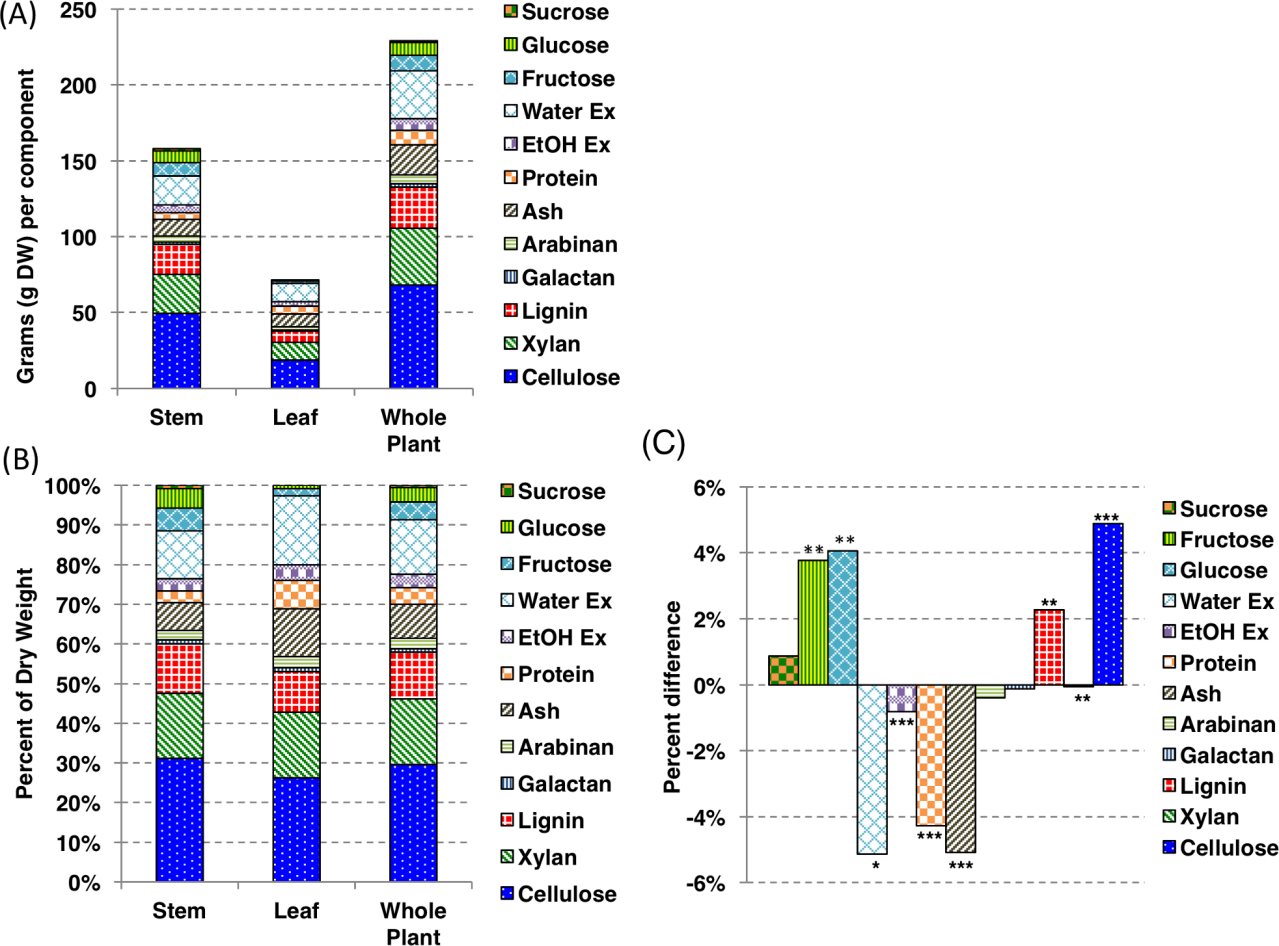
Biomass and composition of TX08001 stems and leaves at 120 DAE. (A) The dry biomass of stems, leaves, and shoots and their components of TX08001 at 120 DAE in 2009 (experiment 2). (B) The percentage of total stem biomass represented by each component as determined by NIRS. (C) Composition differences between TX08001 stem and leaf tissues at 120 DAE. Values indicate the proportion in stem tissue relative to leaf tissue. (*) indicates statistically significant difference (* = α < 0.05, ** = α < 0.01, and *** = α < 0.001) between stems and leaves at 120 DAE. The analysis consisted of 9 biological replicates. Statistics calculated using a t-test. Ex; extractives.

The energy sorghum hybrid Tx08001 grown under field conditions was harvested at 30 day intervals in 2009 to characterize changes in biomass accumulation and composition during ~180 days of crop development [34]. During this time TX08001 remained in the vegetative phase. The current study analyzed TX08001 stem biomass accumulation and composition starting at 90 DAE, a point in time when sufficient biomass had accumulated for potential harvest (~15–20 Mg/ha). Stem dry biomass of TX08001 plants doubled every thirty days from 90–150 DAE under irrigated conditions and the dry biomass of the leaves increased approximately 2-fold during this same time interval [34]. The total above-ground dry biomass of Tx08001 plants at 120 DAE was ~225 g (~30 Mg DW/ha) (Fig 3A). At 120 DAE the average dry weight of the energy sorghum hybrid stems was ~160 g per plant (~21 Mg DW/ha) which accounted for ~66% of the total above ground dry biomass. The dry biomass of the leaves at 120 DAE was ~70 g per plant (~9 Mg/ha) and accounted for ~ 33% of the biomass of the total above-ground dry biomass. The rate of stem biomass accumulation slowed from 150 to 180 DAE [34] and the biomass accumulated during the last month of the season was due primarily to an increase in the sucrose content of the stem.

### TX08001 stem and leaf composition

Stem and leaf biomass was collected from TX08001 plants at 120 DAE for compositional analysis using NIRS (Fig 3). Cell wall polymers (cellulose, GAX, lignin) accounted for ~60% of TX08001 stem biomass and ~55% of leaf biomass at this stage of development (Fig 3B). Cellulose was the most abundant constituent of energy sorghum leaves and stems at 120 DAE, accounting for ~31% of the stem dry biomass and ~25% of leaf dry biomass (Fig 3B). GAX is the major hemicellulose present in grass cell walls. The NREL NIRS model reports on the predicted percentage of arabinose, and xylose that are likely derived from GAX. Considering this, the NIRS data shows that GAX (xylose and arabinose) was the second most abundant polymer, accounting for ~18% and ~20% of the total dry biomass in the stem and leaves respectively (Fig 3B). Lignin was ~12% of stem biomass and ~10% of leaf dry biomass. Arabinose and galactose was present in comparably small quantities (1–3%) in both stems and leaves. Leaf cell walls contained slightly more xylose than stem cell walls (28.4% versus 26.5%).

Approximately 40% of the stem biomass was comprised of nonstructural carbohydrates (glucose, fructose and sucrose), protein, ash, and other extractives (water- and ethanol-soluble). Leaf biomass was ~7% protein, ~2-fold greater than the stem (Fig 3B and 3C). Water extractives not including sugars accounted for ~12% of stem biomass and 17% of leaf biomass (Fig 3B). Ethanol extractives, which include chlorophyll, waxes, and other minor components, were 4% of leaf dry biomass and 3% of the stem dry biomass (Fig 3B). Non-combustible biomass (ash) including silica (non-soil, plant derived) accounted for ~7% and ~12% of the dry biomass of stems and leaves respectively (Fig 3B). As the stem accounted for ~66% of the total plant dry biomass, the whole plant composition more closely resembled the composition of the stem (Fig 3A and 3B).

### Stem cell wall and lignin composition of TX08001 and Della

Stem cell wall composition of TX08001 grown in the vegetative phase for 150 days under field conditions and Della stems harvested just before grain maturity were analyzed in greater depth. TX08001 stem cell walls excluding protein and ash were comprised of 48% cellulose, 32% GAX and 20% lignin, a compositional profile very similar to that of sugarcane (S1 Table)[43]. Cell wall GAX was composed of 26.1% xylose, and 3.4% arabinose (S1 Table).

Analysis of lignin composition by HSQC NMR showed a consistent **S**/**G** ratio of 0.53–0.58 in the cell walls of stems of the energy sorghum hybrid TX08001 and the sweet sorghum Della (Table 1 and S2 Fig). There was more tricin **T** (a chain-initiating unit only recently discovered to be a lignin monomer [44,45]) in Della than in TX08001 (Table 1 and S2 Fig), but more units **A** and **B** containing chain-propagating units identified by their characteristic inter-unit linkages (β–O–4 and β–5) in TX08001. With a lower chain-initiating tricin level in TX08001, we hypothesized that other indicators of chain initiation, ferulate **FA**, or components relating to monolignol dimer formation **C** or **C'**, and those with cinnamyl alcohol signatures **X1**, **Ca** (and perhaps the **Ba** derived from them), would be higher but in fact all appeared to be the same or lower in TX08001 (Table 1 and S2 Fig). Incidentally, as recently reported for maize [44], sorghum has essentially no resinol **C** levels, with all such dimerization coming from sinapyl *p*-coumarate that results in structures **C'** in the lignin. The higher relative β-ether **A** level but the lower chain initiation levels make predicting the relative ease of lignin polymer degradability complex challenging. Additionally, we are looking at average compositions across harvested stem cell types here (because of the homogenization/grinding), so changes in physical structure and lignin distribution could have different (and opposite) impacts. The phenolic acid pendent groups (*p*-coumarate and ferulate, acylating either polysaccharides or lignin) were found to be in higher relative abundances in Della than in TX08001 (S2 Fig).

**Table 1:** Relative levels, determined from 2D-NMR volume-integrals, of various units in the whole-cell-walls of sweet sorghum Della and the energy sorghum TX08001.

	Della (sweet)	TX08001 (energy)
%S^*^	38	36
%G^*^	62	64
%H^*^	3	2
%T (tricin)^*^	3	2
%Ba (benzaldehyde)^*^	5	5
%Ca (cinnamaldehyde)^*^	4	4
%X1 (cinnamyl alcohol)^*^	4	4
**S/G**	0.58	0.53
***p*CA/(S+G)**	0.51	0.44
**FA/*p*CA**	0.63	0.62
%A^**^	75	79
%B^**^	6	8
%C^**^	19	13
**A/OMe**	0.26	0.29

**S**; syringyl, **G**; guaiacyl, ***p*CA**; *p*-coumarate, **FA**; ferulate, β-ether units **A** + **A'** (β–O–4 lignin linkage), phenylcoumarans **B** + **B'** (β–5 lignin linkage), furans **C** + **C'** (β–β lignin linkage), and methoxy, **OMe**.

*Percentages on a **G**+**S**+**S'** = 100% basis

**Percentages on an **A**+**A'**+**B**+**B'**+**C**+**C'** = 100% basis. The data represent the means of two technical replicates of two biological replicates from experiments 2 and 3.

**Fig 4:**
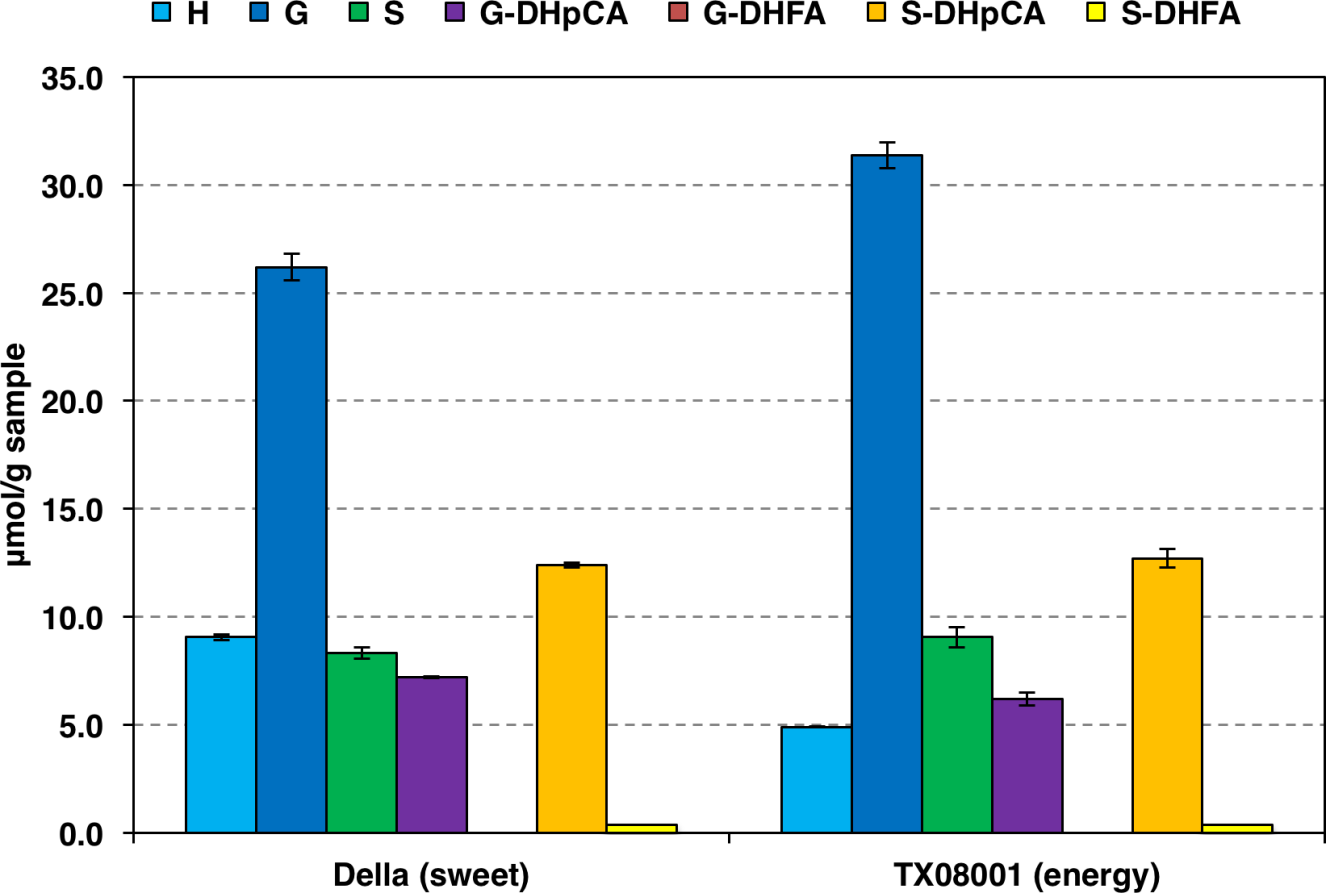
Monolignol and monolignol hydroxycinnamate conjugate concentrations released from TX08001 and Della stems by DFRC. Error bars represent standard error of mean. **H**; 4-hydroxycinnamyl alcohol, **G**; coniferyl alcohol, **S**; sinapyl alcohol, **G-DH*p*CA**; coniferyl dihydro-*p*-coumarate, **G-DHFA**; coniferyl dihydroferulate, **S-DH*p*CA**; sinapyl dihydro-*p*-coumarate, **S-DHFA**; sinapyl dihydroferulate, all as their diacetates. Data were obtained from two technical replicates of two biological replicates for both TX08001 and Della (experiments 2 and 3).

Lignin composition, as determined by DFRC, was not substantially different between Della and TX08001 (Fig 4). As noted by NMR, the amounts of phenolic esters are higher in Della than TX08001. The phenolic acids in sorghum cell-walls are linked to the arabinosyl subunits of the hemicelluloses and to the γ-OH of lignin side-chains. Cell-wall-bound ferulate has been shown to function as a powerful cell wall cross-linking agent, covalently crosslinking hemicelluloses to each other and to lignins [19,46]. Ferulate has also been recently found to acylate lignins, again on the γ-OH of lignin side-chains; as for *p*-coumarates, this lignin acylation results from the pre-acylated lignin monomers [42]. The involvement of monolignol ferulates in lignification produces so-called ‘Zip-lignins’ that are of significant interest because, unlike ‘normal’ lignins, these have ester linkages in the lignin backbone that are readily cleaved during pretreatment processes and therefore reduce the recalcitrance of lignin to processing [47,48]. The increase of ferulate pendent groups in Della indicates a possible increase in ferulate-based crosslinking and/or zip-lignin formation. Detection of the products of ferulate’s crosslinking into lignin is extremely difficult due to the large number of products possible (see S2 Fig Wilkerson *et al*.) [48]. However, DFRC degradation of zip-lignins releases a diagnostic marker compound; the release extent has been shown to be linearly correlated with the amount of monolignol ferulate used to prepare artificial maize cell wall lignins [42]. As there was no apparent change in DFRC-releasable monolignol ferulates, the difference in cell-wall bound ferulate units can be attributed to changes in the ferulate pool on hemicelluloses.

### Variation in stem composition during vegetative growth

**Fig 5:**
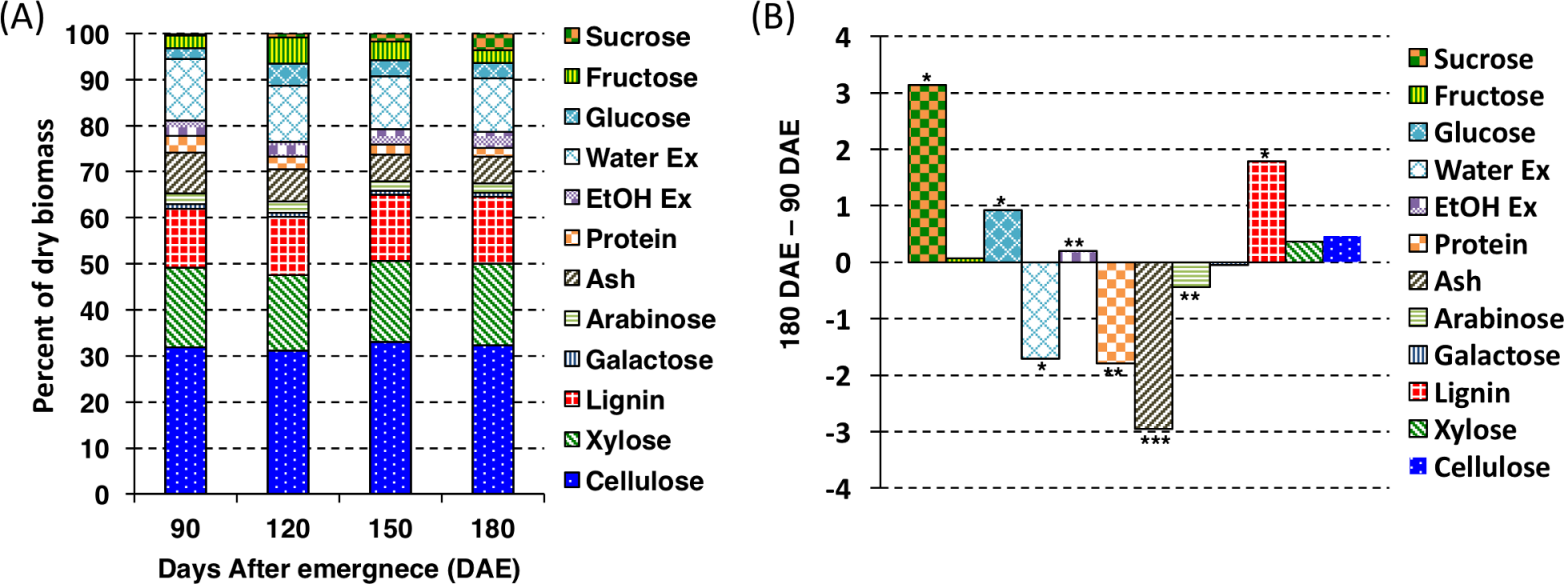
Variation in stem biomass and composition of TX08001 during vegetative development. (A) TX08001 stem biomass components as a percentage of total biomass of TX08001 in 2009 (experiment 2). (B) Composition changes in stem tissue from 90 to 180 DAE of TX08001. Positive values indicate a higher relative percentage in stems at 180 DAE vs. 90 DAE. (*) indicates statistically significant difference (* = α < 0.05, ** = α < 0.01, and *** = α < 0.001) calculated using a t-test, between 90 DAE and 180 DAE component percentages. The analysis consisted of 9 biological replicates. Statistics calculated using a t-test. Ex; extractives.

The composition of TX08001 stems changed to a small extent during crop development from 90 to 180 DAE (Fig 5A). Glucose and fructose as a percentage of the stem dry weight peaked at 120 DAE and declined slightly thereafter. Sucrose as a percentage of stem dry biomass increased from ~0.5% to ~3% during this phase of development (Fig 5B and S3 Fig). Glucose and fructose concentrations peaked at 120 DAE and declined thereafter (S3 Fig). Protein levels in stem biomass gradually decreased from 3.6% to 1.8% during development and water extractives decreased by a similar amount (Fig 5B). Between 90 DAE and 180 DAE, the percentage of the dry biomass that was ash declined by ~3%, from 8.8% to a minimum of ~5.8% (Fig 5A and 5B). The cellulose, lignin, and xylan composition of the cell wall did not change significantly during crop development (S1 Table).

### Water deficit decreases biomass and increases stem sucrose

**Fig 6:**
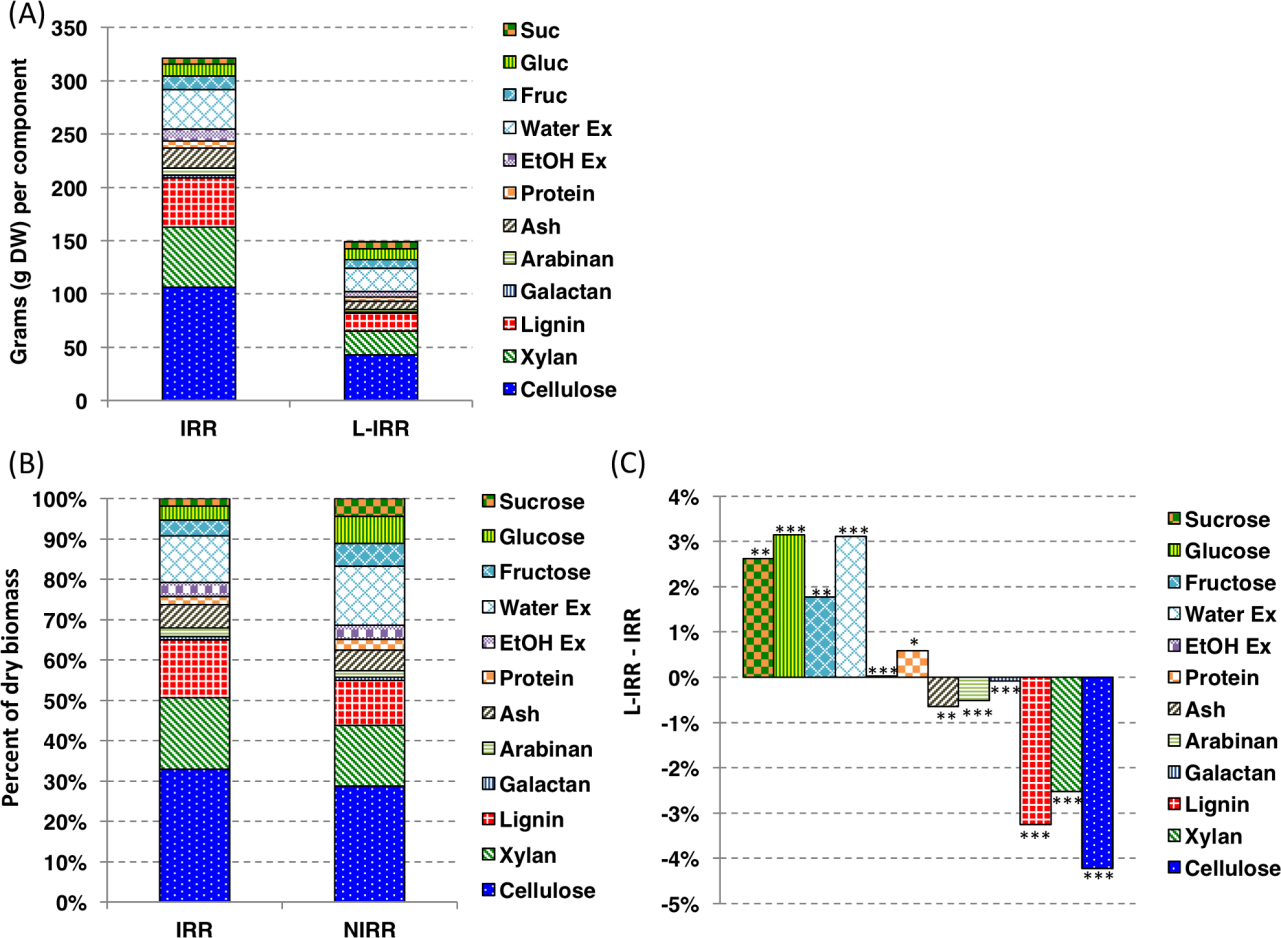
Impact of limited irrigation on the biomass and composition of TX08001 stem tissue at 150 DAE. (A) The difference in dry biomass of irrigated (IRR) and limited irrigation (L-IRR) stems of TX08001 at 150 DAE in 2009 (experiment 2). (B) TX08001 stem biomass components as a percentage of the total composition of irrigated and non-irrigated plants at 150 DAE. (C) Stem composition changes between irrigated and non-irrigated TX08001 at 150 DAE. (*) indicates statistically significant difference (* = α < 0.05, ** = α < 0.01, and *** = α < 0.001) calculated using a t-test, between L-IRR and IRR component percentages. The analysis consisted of 9 biological replicates. Statistics calculated using a t-test. Ex; extractives.

Energy sorghum will routinely be grown without irrigation in regions subject to intermittent periods of water deficit during long growing seasons [3]. Therefore, the impact of water deficit on energy sorghum biomass accumulation and composition was evaluated by growing TX08001 with and without irrigation from July 7 through September 7, 2009 (~150 DAE), summer months nearly always subject to limited rainfall in central Texas [33]. In 2009, between July 7 and harvest in early September significant rainfall (~40 mm) occurred only once on July 25th. Energy sorghum grown with irrigation accumulated ~325 g of stem biomass by harvest, versus ~150 g of stem biomass in plants without irrigation (Fig 6A). In general, the relative amounts of soluble compounds in stems of non-irrigated TX08001 increased relative to cell wall biomass compared to plants grown with irrigation (Fig 6C). The relative amounts of sucrose, glucose and fructose were ~2.6%, ~3.2%, and ~1.7% higher in stems of non-irrigated plants (Fig 6B and 6C). The total non-structural carbohydrate content of irrigated plants was ~9% of the stem’s dry weight whereas non-structural carbohydrates accounted for ~17% of stem dry weight in non-irrigated plants. Metabolites in the water-extractable fraction also increased from 12% to 15% of dry weight in non-irrigated plants (Fig 6B and 6C, p-value >0.001). The amount of protein as a percent of stem biomass in non-irrigated plants was 50% higher than irrigated plants whereas the percentage of the dry biomass allocated to ash decreased slightly from 6% in the irrigated versus 5% in the non-irrigated cohort (Fig 6B).

## Discussion

In the early 1980s, following the oil embargo, forage sorghum’s potential utility as a biofuel crop was evaluated and late flowering genotypes with high biomass yield were identified as promising for high biomass energy crop development [13]. Following a hiatus of nearly 20 years, research on energy sorghum was reinitiated in the late 1990s following the discovery of a feasible way to produce energy sorghum hybrids [49]. Subsequent development of this breeding system established genetic resources useful for production of photoperiod sensitive energy sorghum hybrids with high biomass yield [13,23,33]. This activity also created populations and diversity panels for research aimed at improving the yield, resilience, and composition of energy sorghum [13,50,51].

The composition of biomass has a significant impact on the logistics of harvesting, transport, storage, and the methods, efficiency, and the cost of conversion of biomass to biofuels and bioproducts [10,52]. Composition also impacts potential end-product use and the spectrum of specialty bioproducts that can be produced economically from biomass feedstocks. Techno-economic analysis indicates that the cost of biomass contributes ~38% and conversion to products ~25% to the cost of lignocellulosic biofuels [10]. Therefore, it would be ideal to improve biomass yield and optimize biomass composition while increasing crop resilience.

Due to the relatively recent development of energy sorghum hybrids, there are gaps in our knowledge of the crop’s biomass composition. Prior studies characterized the composition of biomass derived from grain, forage, and high biomass sorghum crops harvested at the end of each crop’s typical growing season [28]. The time-course of sucrose accumulation in sweet sorghum stems has also been characterized [32], as well as the composition of sorghum grain [53]. In the current study, the composition of energy sorghum leaves and stems was characterized during the ~180 day growing season. During development, energy sorghum produces a canopy that closes between ~60–75 DAE [34], followed by rapid stem growth until ~150–175 DAE under optimal growing conditions [33]. This study evaluated the composition of energy sorghum starting at 90 DAE when biomass yield could justify harvesting in some systems of production. Floral initiation occurred in mid-September in the field location used in this study when day lengths decrease below ~12.4 hours after ~150 days of vegetative growth.

First-generation energy sorghum hybrids such as TX08001 have the potential to accumulate >40 Mg/ha of harvestable biomass during 180–210 days of development in good growing conditions if water supply is not limiting [23,33]. Stems account for ~80% and leaves ~20% of harvestable above ground biomass with roots adding another 15–20% to total plant biomass accumulation [33]. Green leaf area reaches a plateau by mid-season when lower leaf senescence matches production of new green leaf area at the top of the canopy [34]. Although leaves account for only ~20% of harvestable biomass, leaf biomass is potentially a significant source of protein and energy that could be separated from stems during harvesting and used for forage [54], processed separately, or returned to the field as a soil amendment [55]. Leaves of energy sorghum have high dry/fresh weight ratios (~0.43) and more protein and ash content compared to stems. Ash (wall-associated + non-wall-associated inorganics) neutralizes dilute-acid pretreatment solvents and therefore reduces its efficiency, increases slag formation during combustion, reduces the efficiency of catalysts, and generates corrosive byproducts resulting from pyrolysis [56]. Leaf biomass is easier to dry compared to stem biomass and ensilage allows long-term storage [57]. Therefore, use of energy sorghum leaf biomass as forage could enhance economic sustainability [54].

Energy sorghum is principally a stem biomass crop, even though panicles and grain may accumulate in some genotypes before harvest in locations where short days induce flowering before harvest. Stem cell walls of TX08001 are composed of ~50% cellulose, ~30% GAX, and 20% lignin and make up the largest fraction of stem biomass (~30–60% of total). The composition of TX08001 stem cell walls was similar to that of other C4 grasses such as sugarcane [58]. The ratio of the major constituents of stem cell walls as well as xylose and arabinose content was relatively constant from 90 DAE to 180 DAE. The composition of Miscanthus stem cell walls also showed minimal variation during the growing season [22].

Sorghum completes elongation of a stem internode approximately every 4 days during vegetative growth and after floral initiation until a week before anthesis. The current study indicates that stem nodes/internodes produced during the growing season are similar in overall cell wall composition. Analysis of DFRC-released monolignol composition showed that TX08001 stem lignin has a ratio of syringyl (**S**) to guaiacyl (**G**) units of approximately 0.53–0.58 (**S**/**G**), similar to that of other C4 grasses. Increasing sorghum stem lignin **S**/**G** ratios may improve saccharification efficiency as has been demonstrated in other plants [59,60]. For the hydroxycinnamates, *p*-coumarate (***p*CA**) and ferulate (**FA**), the amount of ***p*CA**/(**S**+**G**) and **FA**/***p*CA** ratios differ significantly between stems of the sweet sorghum Della and TX08001; these differences were related to the change in hemicellulose-bound ***p*CA** and **FA** and not changes the lignin bound ***p*CA** and **FA**. The biological significance of these differences in cell wall composition has not been examined, but indicates that there is significant natural variation in the extent and chemistry of cell wall crosslinking that might provide useful ways to improve biomass saccharification [48]. Changes in cell wall cross-linking may also improve forage digestibility in conjunction with variation in lignin composition and chemistry already available in forage sorghum *bmr*-genotypes [61–63]. Recent development of new methods for biomass and lignin deconstruction, removal, and conversion to useful products may significantly improve lignin utilization while increasing the accessibility of other cell wall constituents for conversion to biofuels and bio-products [64–66].

Sorghum stems can accumulate up to 50% of their biomass in the form of sucrose, glucose and fructose [4,32]. In addition to fermentable sugars, the stem’s soluble components also include protein, MLG, ash and a diverse set of other compounds. Efficient utilization of these heterogeneous materials will enhance the economics of energy sorghum production. The proportion of soluble biomass relative to structural biomass in stems of diverse energy sorghum genotypes ranged from 55% to 15%. Accumulation of stem sugars in genotypes that had reached floral initiation was correlated with higher levels of soluble biomass. The high levels of fermentable sugars in energy sorghum stems could be extracted and converted to biofuels and bioproducts at relatively low cost and high efficiency. This suggests that one viable approach to improving energy sorghum biomass is to increase the amount and density of non-structural carbohydrates in energy sorghum stems. Energy sorghum genotypes with minimal levels of soluble stem biomass were also identified (~15% of total). Interestingly, there was little correlation between the dry/fresh weight ratios and the ratio of soluble/structural biomass.

Crop residues and perennial bioenergy grass crops such as switchgrass and Miscanthus are harvested at the end of the season after plants have senesced following remobilization of a significant portion of their carbon and nitrogen to roots for use the following season. Although remobilization reduces biomass yield by ~20–30% the resulting biomass has a higher dry/fresh weight decreasing transportation costs and increasing biomass stability. In contrast, energy sorghum and sugarcane are typically harvested prior to senescence when canopies are green and sucrose levels in stems are high. Energy sorghum stems have high moisture content (~70–85%) at harvest, similar to sugarcane. Sorghum stems dry slowly after harvest due to their structure and high amounts of surface wax, especially in regions of production that have high relative humidity. Differences in cell wall content/unit stem volume, thickness, or the formation of stem aerenchyma could contribute to the observed variation in stem dry/fresh weight [67,68].

The utility of large high-moisture stems of sorghum and sugarcane that have the capacity to accumulate sugars has been recently reviewed [4]. The stems of sugarcane and sorghum can accumulate ~0.5 M sucrose [4]. Sucrose, glucose and fructose can account for 50% of sweet sorghum stem dry weight [32] providing significant buffering when the demand for carbohydrate for growth or seed development is low relative to photosynthetic activity. The accumulated sugars are a source of carbohydrate for seed filling post-anthesis, tiller production after grain maturation, or for growth following periods of adverse weather during the vegetative phase. In the current study TX08001 accumulated higher levels of stem sugars during the vegetative phase under water-limiting conditions when cell wall biomass accumulation associated with growth was reduced.

## Supporting information

S1 Fig A comparison between stem nonstructural carbohydrate profiles, dry to fresh biomass ratios, and the percentage of soluble and structural biomass from a representative selection of energy and sweet sorghums. Data were obtained from plant material from experiments 1, 2, and 3. ESAP samples consisted of bulked internodes taken from five plants that were harvested at 150 DAE (experiment 1). Data from TX08001 (experiment 2) and Della (experiment 3) were obtained from 9 plants harvested at 150 DAE and bulked into three samples. (A) Stem nonstructural carbohydrtes profiles from select energy and sweet sorghums. Measurement of sucrose, glucose, fructose and starch was performed in duplicate and MLG assays were performed in triplicate. Error bars represent standard error of the mean. (B) Ratio of dry biomass to fresh biomass of sorghum internodes at 150DAE from the sorghum panel described above. (C) NIRS prediction of the percentage of the sorghum stem dry biomass that is composed of soluble and structural molecules from the panel described above. Genotypes with * flowered during the experiment. Each bar represents data obtained from five bulked internode segments from ESAP accessions.
(TIFF)

S2 Fig 2D ^1^H–^13^C HSQC NMR spectra of energy sorghum stem cell walls at 150 days after emergence and sweet sorghum stem cell walls at anthesis. (A, B) Aromatic region, percentages are based on the summation of peak area of **G** + **S** = 100. (C, D) Aliphatic region, percentages based on summation of the area of the side chain signals for the three components, **A** + **B** + **B'** + **C'** = 100%.
(TIFF)

S3 Fig Time-course of nonstructural carbohydrate accumulation during energy sorghum TX08001 development. Data were obtained from plant material harvested form irrigated TX08001 in 2009. The red data series represents glucose, purple represents fructose, and turquoise represents sucrose. Errors bars represent standard error of mean.
(TIFF)

S1 Table Cell wall composition of the energy sorghum stem determined by NIRS at 60–180 DAE. The data were obtained from Tx08001 field grown plants in 2008. To measure variation in cell wall composition throughout the growing season, the means of all time-points of each trait were used to calculate the standard deviation for that trait through time.
(DOCX)
