# Supplementary figures and images for: Variation in energy sorghum hybrid TX08001 biomass composition and lignin chemistry during development under irrigated and non-irrigated field conditions
Source: PLoS One. 2018 Apr 23;13(4):e0195863. doi: 10.1371/journal.pone.0195863 (PMC5912772; doi:10.1371/journal.pone.0195863)

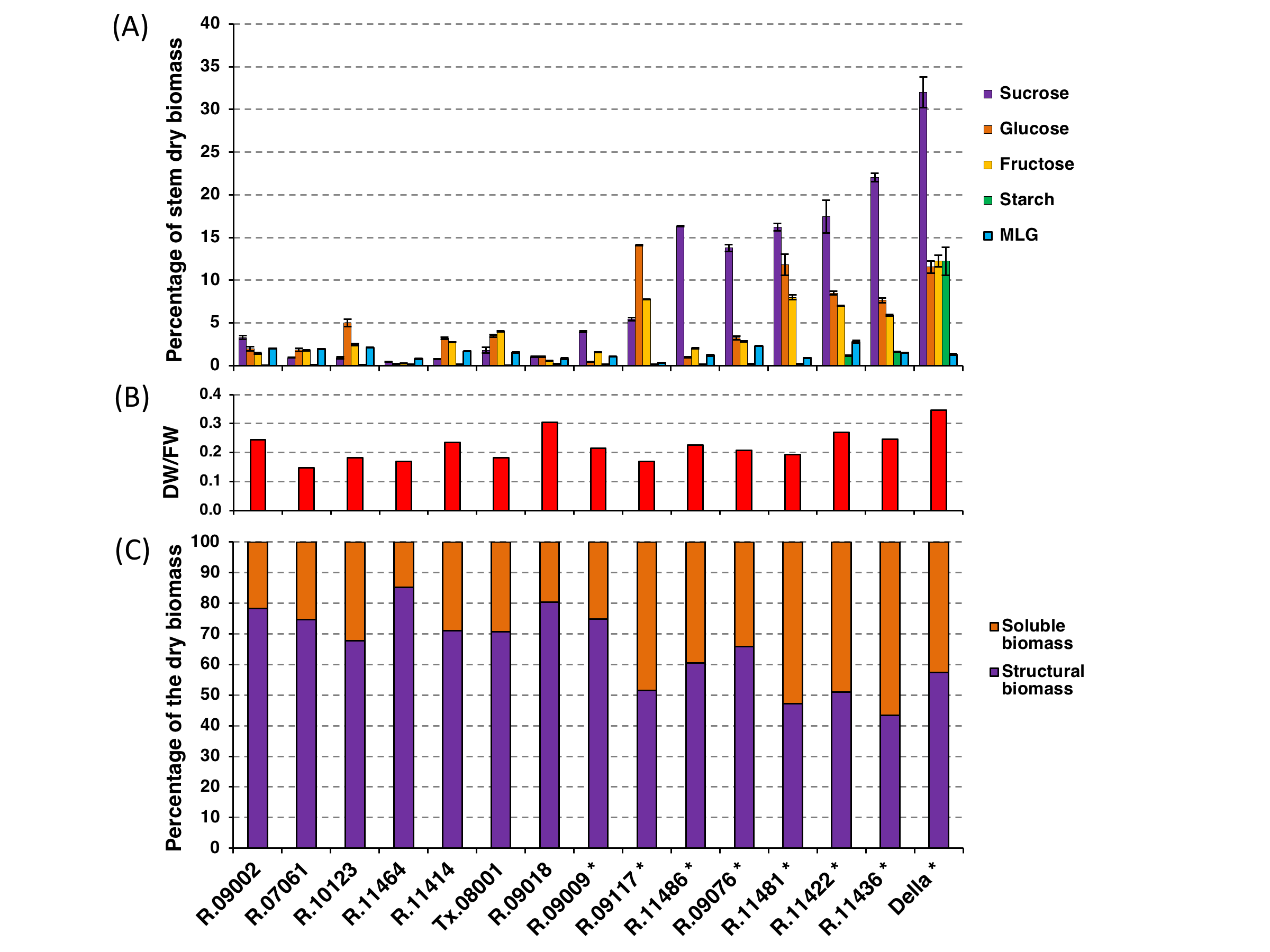

Supplement: S1 Fig — Data were obtained from plant material from experiments 1, 2, and 3. ESAP samples consisted of bulked internodes taken from five plants that were harvested at 150 DAE (experiment 1). Data from TX08001 (experiment 2) and Della (experiment 3) were obtained from 9 plants harvested at 150 DAE and bulked into three samples. (A) Stem nonstructural carbohydrtes profiles from select energy and sweet sorghums. Measurement of sucrose, glucose, fructose and starch was performed in duplicate and MLG assays were performed in triplicate. Error bars represent standard error of the mean. (B) Ratio of dry biomass to fresh biomass of sorghum internodes at 150DAE from the sorghum panel described above. (C) NIRS prediction of the percentage of the sorghum stem dry biomass that is composed of soluble and structural molecules from the panel described above. Genotypes with * flowered during the experiment. Each bar represents data obtained from five bulked internode segments from ESAP accessions. (TIFF) [file pone.0195863.s001.tiff]

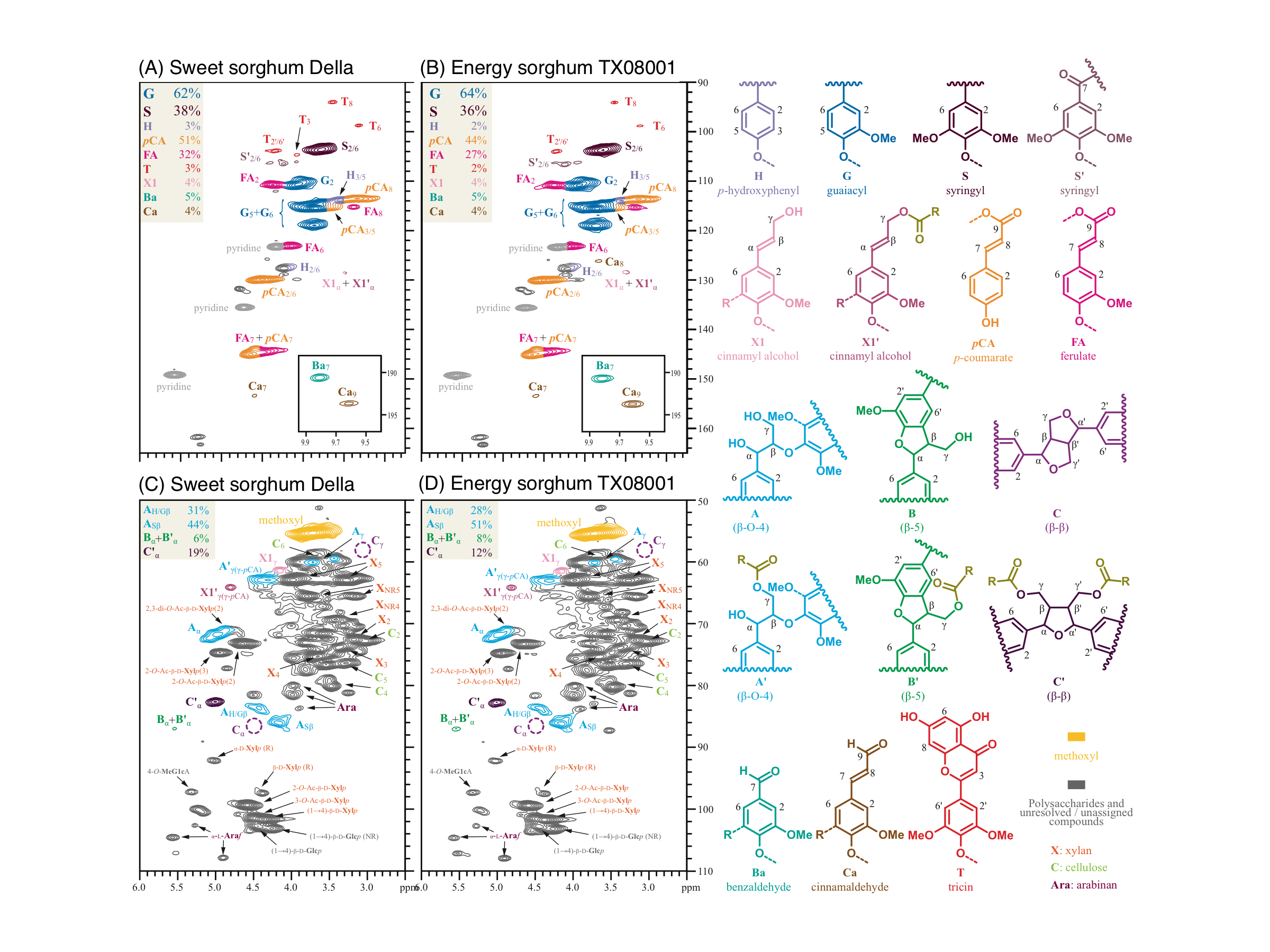

Supplement: S2 Fig — (A, B) Aromatic region, percentages are based on the summation of peak area of G + S = 100. (C, D) Aliphatic region, percentages based on summation of the area of the side chain signals for the three components, A + B + B' + C' = 100%. (TIFF) [file pone.0195863.s002.tiff]

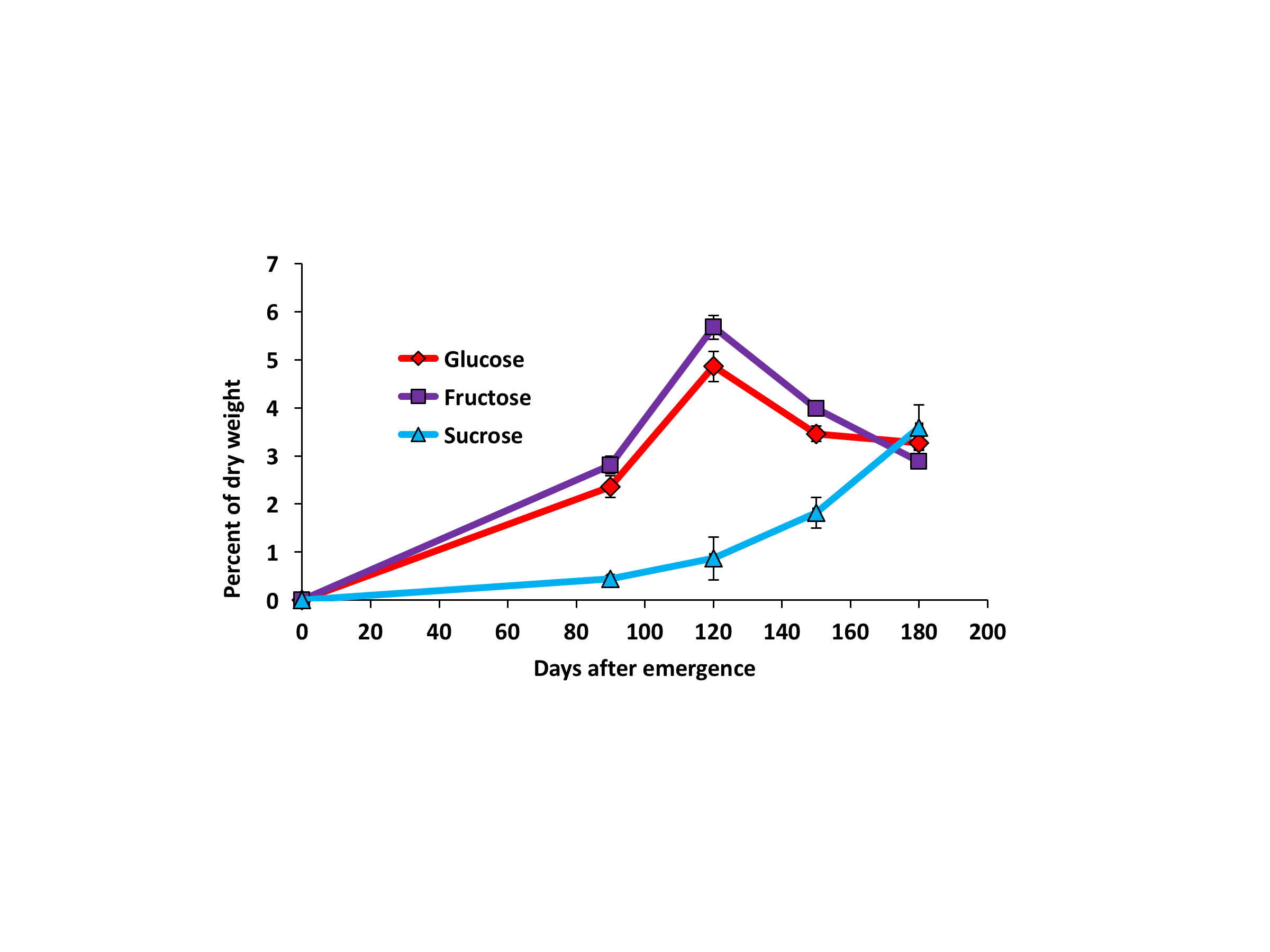

Supplement: S3 Fig — Data were obtained from plant material harvested form irrigated TX08001 in 2009. The red data series represents glucose, purple represents fructose, and turquoise represents sucrose. Errors bars represent standard error of mean. (TIFF) [file pone.0195863.s003.tiff]
