# Supplementary material for: Variation in energy sorghum hybrid TX08001 biomass composition and lignin chemistry during development under irrigated and non-irrigated field conditions
Source: PLoS One. 2018 Apr 23;13(4):e0195863. doi: 10.1371/journal.pone.0195863 (PMC5912772; doi:10.1371/journal.pone.0195863)
Supplement: S1 Table — The data were obtained from Tx08001 field grown plants in 2008. To measure variation in cell wall composition throughout the growing season, the means of all time-points of each trait were used to calculate the standard deviation for that trait through time. (DOCX) [file pone.0195863.s004.docx]

**S1 Table. Cell wall composition of the energy sorghum stem determined by NIRS at 60-180 DAE.**

| **Leaves** | **Cellulose %** | **Xylose %** | **Lignin %** | **Arabinose %** | **Galactose %** |
| --- | --- | --- | --- | --- | --- |
| **60 DAE** | 47 | 30 | 18 | 3.7 | 1.6 |
| **90 DAE** | 45 | 28 | 20 | 5.2 | 1.8 |
| **120 DAE** | 49 | 29 | 15 | 5.1 | 2.0 |
| **150 DAE** | 48 | 28 | 16 | 5.8 | 2.0 |
| **180 DAE** | 47 | 27 | 18 | 5.5 | 2.0 |
| **StDev:** | ± 1.6 | ± 1.1 | ± 1.9 | ± 0.8 | ± 0.18 |
|  |  |  |  |  |  |
| **Stems** | **Cellulose %** | **Xylose %** | **Lignin %** | **Arabinose %** | **Galactose %** |
| **60 DAE** | 48 | 26 | 20 | 4.4 | 1.6 |
| **90 DAE** | 47 | 26 | 21 | 3.8 | 1.7 |
| **120 DAE** | 49 | 27 | 19 | 3.8 | 1.8 |
| **150 DAE** | 49 | 26 | 19 | 3.4 | 1.6 |
| **180 DAE** | 49 | 26 | 19 | 3.5 | 1.8 |
| **StDev:** | ± 1.1 | ± 0.2 | ± 0.9 | ± 0.4 | ± 0.1 |
